# Editorial: Autonomous weed control for crop plants

**DOI:** 10.3389/fpls.2024.1527610

**Published:** 2024-12-09

**Authors:** Wen-Hao Su

**Affiliations:** Department of Agricultural Engineering, College of Engineering, China Agricultural University, Beijing, China

**Keywords:** artificial intelligence, agricultural robots, computer vision, control systems, sensors

Weed infestation has always influenced agricultural production. Weeds in agricultural fields have a strong ability to reproduce, and they compete with crops for resources such as nutrients, water and light, directly or indirectly affecting the yield and quality of crops. In order to reduce the loss of crop production, timely and effective control of weed is particularly important. Manual weeding can accurately remove weeds, but it is labor-intensive, costly. Mechanical weed control methods are challenging because of the high risk of damage to crops when removing weed in close proximity to them. Therefore, more efficient and advanced weed control technologies have become an essential part of modern agriculture. This Research Topic aims to explore recent advanced weed control technology for crop plants for real world applications.

In this Research Topic, a total of 7 papers have been published, encompassing the contributions of 40 distinct authors. The content primarily delves into the realms of advanced weed detection, localization and weed control technology based on existing foundations.

Andreasen et al. investigated the use of laser technology for weed control, focusing on the effectiveness of laser irradiation at different growth stages of common weeds, particularly in the early growth stages. The study found that laser weeding was most effective on young weeds (cotyledon to two-leaf stage), offering a non-chemical alternative to traditional herbicides and addressing the issue of herbicide resistance. This non-chemical method not only reduced the reliance on herbicides but also provided a viable solution for managing herbicide-resistant weeds, marking a significant step towards more sustainable weed control.

Li J. et al. developed a real-time smart sensing system for the automatic localization and recognition of vegetable plants,aiming to enhance the accuracy and reliability of autonomous weed control. The system used a combination of cameras and color mark sensors to accurately identify and locating plants, achieving an overall accuracy of 95.,19%. The proposed system is highly reliable and effective for precise weed control in vegetable crops, especially in the early stages of crop growth.

Xie et al. developed an intelligent spraying system for wheat fields to solve problems including the low herbicide adhesion rate, which optimized the application of herbicides, to improve the precision and efficiency of weed control. By using a dynamic model of droplet impact on wheat leaves, this system enhanced crop safety and reduces environmental impact, contributing to more precise and efficient herbicide use. The research identified optimal spraying parameters (0.4 MPa pressure, 0.011 kg/s flow rate at 300 mm height) to maximize herbicide adhesion and minimize splash, achieving precise and efficient weed control.

Wang et al. utilized the Swin Transformer and a two-stage transfer learning strategy to develop a fine-grained weed recognition method. The method effectively distinguished subtle differences between visually similar weeds and crops, achieving high accuracy and robustness. The study demonstrated superior performance compared to state-of-the-art CNN-based architectures, using a private dataset and the public Deep Weeds dataset.

Xiang et al.provided a comprehensive review of crop detection technologies and mechanical weeding components used in intelligent mechanical weeding systems, identifying the strengths and limitations of current technologies and highlighting the need for interdisciplinary collaboration to improve the performance and reduce the costs of these systems. This review served as a valuable resource for researchers and practitioners, guiding the development of more efficient and reliable weeding systems, making them more accessible for widespread adoption.

Li J. et al. introduced a local path planning a method for orchard mowers, combining safe corridors and quadratic programming to address the challenges of kinematic constraints and unstructured environments. The proposed method used a depth-first search to construct safe corridors and introduced piecewise jerk and curvature restrictions into quadratic programming. This ensured high-order continuity and curvature feasibility, reducing the difficulty of tracking control. The results showed significant improvements in path smoothness and computational efficiency, with an average computation time of 9.6 ms, a 129% improvement over existing methods. It enhanced the operational safety and efficiency of orchard mowers in complex environments.

Li J. et al. evaluated semi-supervised learning frameworks for multi-class weed detection, using FCOS and Faster-RCNN models. By leveraging a small set of labeled data and a large set of unlabeled data, the proposed method achieves high accuracy and efficiency in weed recognition, significantly reducing the need for manual labeling and improving the performance of weed management systems.

These studies collectively present a multifaceted approach to precision agriculture, with each one building upon and supplementing the existing foundations. These innovations are paving the way for a more sustainable, efficient, and environmentally friendly future in agriculture.

